# Effects of Lifelong Musicianship on White Matter Integrity and Cognitive Brain Reserve

**DOI:** 10.3390/brainsci11010067

**Published:** 2021-01-06

**Authors:** Edna Andrews, Cyrus Eierud, David Banks, Todd Harshbarger, Andrew Michael, Charlotte Rammell

**Affiliations:** 1 Linguistics Program, Duke University, Durham, NC 27708, USA; cyrus.eierud@duke.edu; 2 Duke Institute for Brain Sciences (DIBS), Duke University, Durham, NC 27710, USA; andrew.michael@duke.edu; 3 Center for Cognitive Neuroscience, Duke University, Durham, NC 27710, USA; 4 Department of Statistical Science, Duke University, Durham, NC 27708, USA; david.banks@duke.edu; 5 Brain Imaging and Analysis Center (BIAC), Duke University, Durham, NC 27710, USA; todd.harshbarger@duke.edu; 6 World Languages, Minot State University, Minot, ND 58707, USA; charlotte.rammell@minotstateu.edu

**Keywords:** cognitive reserve, musicianship, diffusion tensor imaging, fractional anisotropy, white matter integrity

## Abstract

There is a significant body of research that has identified specific, high-end cognitive demand activities and lifestyles that may play a role in building cognitive brain reserve, including volume changes in gray matter and white matter, increased structural connectivity, and enhanced categorical perception. While normal aging produces trends of decreasing white matter (WM) integrity, research on cognitive brain reserve suggests that complex sensory–motor activities across the life span may slow down or reverse these trends. Previous research has focused on structural and functional changes to the human brain caused by training and experience in both linguistic (especially bilingualism) and musical domains. The current research uses diffusion tensor imaging to examine the integrity of subcortical white matter fiber tracts in lifelong musicians. Our analysis, using Tortoise and ICBM-81, reveals higher fractional anisotropy, an indicator of greater WM integrity, in aging musicians in bilateral superior longitudinal fasciculi and bilateral uncinate fasciculi. Statistical methods used include Fisher’s method and linear regression analysis. Another unique aspect of this study is the accompanying behavioral performance data for each participant. This is one of the first studies to look specifically at musicianship across the life span and its impact on bilateral WM integrity in aging.

## 1. Introduction

Since the appearance of the Bialystok, Craik and Freedman article, “Bilingualism as a protection against the onset of symptoms of dementia” (2007), which reported the protective effects of lifelong bilingualism in the appearance of dementia symptoms and the role of cognitive reserve, there have been a large number of articles devoted to this topic, resulting in a robust set of research that has examined bilingualism and its role in building cognitive brain reserve in healthy subjects and pathology [1]. Related to these studies is a series of analyses that point to the role played by cognitive reserve in musicians, which parallels the work on bilingualism in significant ways. In order to clearly position the research presented in the current paper, we will briefly review the definitions of different types of brain reserve, as well as review the findings across studies that yield consensus and/or dissent. This will include work on cognitive reserve in musicians as an important part of the literature. While normal aging produces trends of decreasing WM integrity, research on cognitive brain reserve suggests that complex sensory–motor activities across the life span may slow down or reverse these trends.

One of the earlier sets of terms proposed in Valenzuela and Sachdev (2006) is “neurological brain reserve” and “behavioral brain reserve” [2]. Neurological (brain) reserve is generally considered to be more biologically and genetically based, and it is articulated in the following way by Stern (2012) and Guzmán-Vélez (2015) [3,4]:

The neurological brain reserve hypothesis proposes that individuals generally differ in the numbers of neurons and synapses available to be lost before clinical symptoms emerge (Stern, 2012). Following Guzmán-Vélez, “Brain reserve refers to ‘passive’ factors (e.g., brain volume, synapse count) that confer a particular capacity to endure neuropathological processes until a critical threshold is reached, after which cognitive and functional impairments are expressed” (Guzmán-Vélez, 2015 [4]).

By way of contrast, cognitive reserve, also called behavioral brain reserve, is acquired through specific sensory–motor activities that span across the life cycle (including but not restricted to musicianship and bilingualism). Bialystok et al. (2017) referred to this type of cognitive brain reserve as “resilience to neural insult” and noted that the development of strategies that strengthen alternative functional neural networks across the life cycle can improve tolerance of atrophy (Bialystok, 2017) [1]. These changes may be observed in gray and white matter. Gold et al. (2013) further suggested that (1) higher cognitive reserve should require more structural decline in order for symptoms to manifest and (2) cognitive reserve is an active form of reserve, described as the ability for plastic functional brain reorganization of cognitive networks in response to injury, aging or disease [5]. For more discussion on cognitive reserve, see Craik et al., 2010; Andrews et al., 2013; Andrews, 2014; and de Bot, 2009 [6,7,8,9]. Bialystok et al.’s definition for cognitive reserve serves as the basis for the definition used in the current paper [1].

In the current work, we will examine white matter (WM) integrity and, in particular, fractional anisotropy (FA) values in healthy subjects who are highly proficient musicians. Fractional anisotropy (FA) is one of the measurements extracted from diffusion tensor imaging (DTI) data and is based on the degree of movement of water molecules (between 0 and 1). Higher FA values indicate higher WM integrity.

### 1.1. Cognitive Reserve in Bilinguals

Pliatsikas et al. (2015) considered different groups of bilinguals based on their age of acquisition, including (a) early bilinguals, (b) late bilinguals, (c) older bilinguals and (d) young late bilinguals [10]. Findings showed that all of these different groups showed increased FA values (compared to monolinguals) across several regions, including the genu, body and anterior part of the splenium of the corpus callosum (CC), extending bilaterally to the inferior frontal-occipital fasciculus (IFOF), the uncinate fasciculus (UF) and the superior longitudinal fasciculus (SLF). Similar findings about the greater FA values in the SLF and UF of bilinguals as opposed to monolinguals were given by Luk et al. (2011) [11]. Anderson et al. (2018) found greater WM integrity and higher axial diffusivity (AD) values in bilinguals across several regions (including the bilateral superior posterior corona radiate, the right external capsule, the midbody and splenium of the CC, the left STLF and the anterior IFOF), while Coggins et al. (2004) and Felton et al. (2017) identified greater WM volume specifically in the CC of bilinguals versus their monolingual peers [12,13,14]. The ICBM-81 atlas used in the current analysis includes the AF within the SLF (Mori et al., 2008; Oishi et al., 2008) [15,16].

The mechanisms for the different types of brain and cognitive reserve are poorly understood but may include enhanced generation of neuronal, dendritic and synaptic connections and functional reorganization of neural networks across multimodalities. For example, Grundy et al. (2017) argued in favor of a shift to subcortical/posterior region-based networks in bilinguals, as opposed to the more frontal regions in monolinguals [17]. Abutalebi et al. (2015) focused on increased gray matter (GM) in bilingualism as an example of cognitive reserve and potential protective effects in aging [18,19].

Thus, while cognitive brain reserve may play an important role in delaying the appearance of the symptoms of certain pathologies, it also may play a role in healthy subjects. That is the focus of our current study—lifelong musicians and the potential effects of musicianship on subcortical WM matter fiber tracts. While we do not consider GM in this paper, we do not exclude the importance of cognitive reserve in GM volume changes (cf. Pliatsikas et al., 2015) [10].

### 1.2. Cognitive Reserve in Musicians

Elmer et al., (2018, 2019) have worked across the aisle on aspects of musicianship and bilingualism, including studies focusing on bilingualism and simultaneous translators (2019) and the relationship of music training with speech processing and lexical acquisition (2018) [20,21]. Elmer and Jäncke (2018) pointed out that interest in professional musicians is not new in 21st-century neuroscience but rather dates back to the mid-20th century, and they included mention of studies using a variety of neuroimaging approaches that analyzed the relationship of musicianship and neuroplasticity, neuroanatomical correlates of musicianship, enhanced encoding of vowels and speech in professional musicians, timbre-specific auditory cortical representations in musicians, shared networks for auditory and motor processing in professional musicians, etc. (e.g., Jäncke, 2009; Munte et al., 2002; Bermudez et al., 2009; Kuhnis et al., 2013; Pantev et al., 2001; Bangert et al., 2006; Weiss and Bidelman, 2010) [20,22,23,24,25,26,27,28].

Two interesting DTI studies that provide a context for the current analysis were conducted by Halwani et al. (2011) and Oechslin et al. (2010) [29,30]. In the work of Halwani et al. two different groups of musicians (vocalists and instrumentalists) demonstrated larger WM tract volume and higher FA values for bilateral arcuate fasciculus (AF) [29]. Additionally, differences were found between instrumentalists and vocalists by subdividing the AF into a dorsal superior connection to the STG and IFG and the ventral inferior branch connecting the MTG and IFG. The results confirmed that, in all cases, the FA values were higher for both types of musicians compared to nonmusicians. As noted above, our analysis uses the ICBM-81 atlas, where the AF is included in the SLF [15,16].

The Oechslin et al., study (2010) examined two groups of musicians (with absolute pitch (AP) vs. relative pitch (RP)) and nonmusicians [30]. They analyzed the FA values of the SLF, since the SLF is considered important in processing both speech and music. Findings included specific differences in the SLF between AP and RP, where there was an additional left hemisphere lateralization of FA values. Oechslin et al. also proposed the Pioneer Axon Thesis, which states that “peripheral WM development (contrary to compact WM core regions) is influenced considerably by environmental factors, in this case musical training spanning a long period in postnatal life” (2010: 9) [30].

Other DTI research involving musicians and music-based treatments includes the UF. Yuskaitis et al., (2015) and Wan et al. (2010) discussed the importance of this tract in pitch perception in healthy subjects and its importance for music therapies in the treatment of various disorders [31,32].

### 1.3. Research Question and Hypotheses

The background review of studies on cognitive reserve, the potential interactions between bilingualism and musicianship and studies specifically focusing on musicianship provided the foundation for our research question and hypotheses. It has been shown in multiple studies that WM integrity decreases in normal aging (cf. Tang et al., 1997; Giorgio et al., 2010; Westlye et al., 2010; Billiet et al., 2015; Rathee et al., 2016) [33,34,35,36,37].

Research interested in cognitive reserve has noted that the process of WM integrity loss can be slowed down or changed by lifelong bilingualism and musicianship. Our hypothesis was that there would be an increase in WM integrity in certain subcortical fiber tracts in lifelong musicians, and that this increase would be reflected bilaterally in FA values for the bilateral superior longitudinal fasciculus (SLF) and the uncinate fasciculus (UF)—two specific WM tracts shown to be relevant in musicianship [29,30,31,32]. We also hypothesized that the bilateral SS, which includes the IFOF (a tract important in bilingualism), may not show the same effects. This is one of the first studies to consider specifically lifelong musicianship and include ages ranging from 20 to 67 years.

## 2. Materials and Methods

### 2.1. Participants

Eight musicians (five female; between the ages of 20 and 67 years, with a mean age of 44.1 years) were scanned in 2019–2020 at Duke University Hospital at the Duke Brain Imaging and Analysis Center. A ninth subject was unable to be included due to incomplete behavioral data. All subjects began musical training between the ages of 3 and 12 years, at an average of 6.4 years. The number of years the subjects had been musicians was a correlate of chronological age since all were currently active musicians (mean 38 years).

All were currently active musicians and performed regularly. Subjects reported an average of 3 h per day rehearsing/practicing and an average of 9 h per day in peak times (i.e., during periods of performance). Additional credentials included college degrees in music (including undergraduate minor, master’s and doctorate) and multiple affiliations with orchestra, symphony or operatic theatre in the United States and abroad. Five of the professional musicians had extensive experience in the United States and abroad. All subjects played at least the piano or violin.

### 2.2. Data

#### 2.2.1. Behavioral Data

All eight participants completed an extensive background questionnaire on their musicianship and knowledge of languages. The musical information included detailed information about their entire musical careers as learners, performers and teachers where relevant, amount of practice and playing from inception, formal musical training and education, musical juries and examinations, enjoyment and aesthetics, family ties to music and musical memories. All participants were required to practice a specific piece by Bach or Mendelssohn each day for 5 days prior to the scan date.

#### 2.2.2. Image Acquisition

All subjects were scanned in the same scanner at Duke University Hospital, using a GE 3T Discovery 750 MRI (General Electric, Milwaukee, WI, USA) with an 8-channel head coil. Diffusion-weighted images (DWIs) were collected using an oblique single-shot spin-echo echo-planar imaging (EPI) sequence with a repetition time (TR) = 9000 ms, echo time (TE) = 93 ms, having a 256 × 256 image matrix over a 25.0 cm^2^ FOV, covering 69 interleaved axial slices, each 2 mm thick. Twenty-five diffusion directions were uniformly distributed in 3D space with a b = 1000 s/mm^2^, together with an acquisition with b = 0 s/mm^2^. A T2 and proton-density-weighted sequence (FRFSE-XL) with a TR = 3000 ms, TE = 90 ms, 256 × 256 matrix, 24.0 cm^2^ FOV and 5 mm slice thickness was used to acquire 28 interleaved axial slices.

#### 2.2.3. DTI Processing

All images were processed using Tortoise 3.1.4 (Pierpaoli et al., 2010; Irfanoglu et al., 2017) and FMRIB Software Library (FSL; Woolrich et al., 2009; Smith et al., 2004; Jenkinson et al., 2012) [38,39,40,41,42]. The raw DTI data were corrected for distortion, eddy currents and motion using the DIFFPREP command in Tortoise and a subject-specific T2 weighted image. A brain mask was then generated from the distortion corrected B0 using ExtractImage and bet2. All Tortoise quality control outputs were inspected for signal dropouts, excessive motion and B0 and T2 registration accuracy. Then, the Tortoise commands EstimateTensorNLLSRESTORE, with the RESTORE option and ComputeFAMap, generated the fractional anisotropy maps. Next, FSL was used to normalize the individual FA maps to MNI space, using the FLIRT and FNIRT commands. Finally, the FA for each ROI in each subject were averaged, using the commands fslmaths and fslstats. Using the ICBM-81 atlas (Mori et al., 2008), FA values in the following regions of interests were computed [15]: the left and right superior longitudinal fasciculus (LSLF and RSLF), the left and right sagittal striatum (LSS and RSS) and the left and right uncinate fasciculus (LUF and RUF).

#### 2.2.4. Statistical Methods

**Figure 1:**
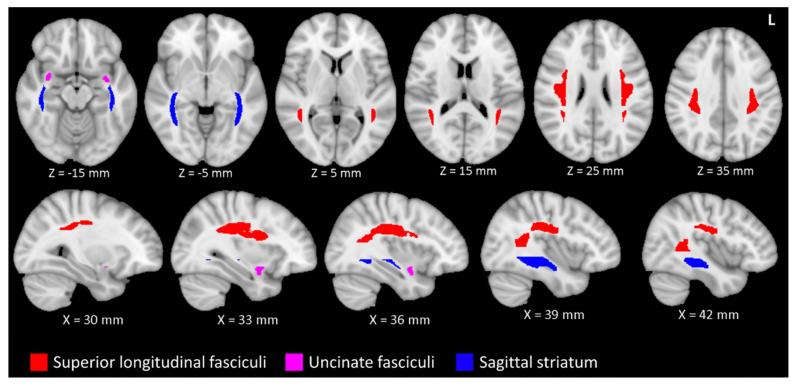
The superior longitudinal fasciculi (SLF) and uncinate fasciculi (UF) are related to musical proficiency and age. The sagittal striata (SS) are also related to age, but not musical proficiency. The SLF (red), UF (magenta) and SS (blue) are depicted in accordance with the ICBM-81 atlas (Mori et al., 2008)[15]. The tracts are superimposed on a T1 weighted image in the Montreal Neurological Institute 152 space. The figure uses the left-posterior-inferior convention. Legend: L, left.

In order to test our hypotheses concerning changes in WM integrity in aging musicians, we used linear regression calculations of the six regions of interest (LSLF, RSLF, LUF, RUF, LSS and RSS), according with Figure 1, followed by an application of Fisher’s method for pooling *p*-values (a statistical method for combining results from multiple tests) [43]. Under the null hypothesis that a regression does not have a slope that is different from 0, the *p*-value on the slope parameter was uniformly distributed between 0 and 1. Fisher (1925) showed that the test statistic

$$-2\overset{k}{\underset{i=1}{\sum }}ln\left(p_{i}\right)$$

has the chi-squared distribution with 2*k* degrees of freedom, where *k* is the number of *p*-values being pooled [43]. The intuition is that if one has several independent experiments, each of which modestly supports the alternative hypothesis, then by combining the weak signals one can increase power for rejecting the null hypothesis.

## 3. Results

**Figure 2:**
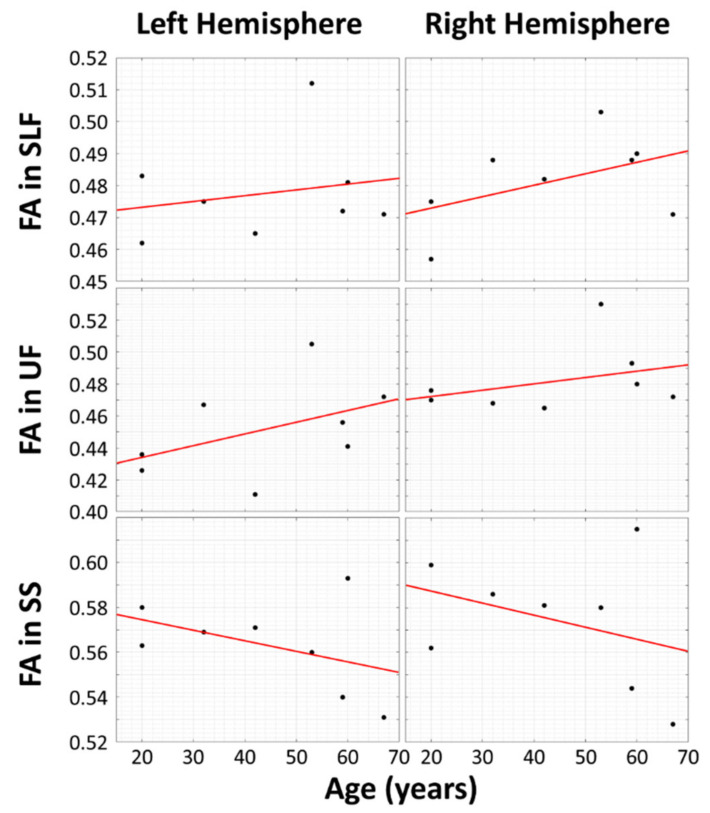
Scatter plots and linear fits between fractional anisotropy (FA) and subjects’ ages that are affected by musical proficiency (top two rows) and tracts that may be unrelated to musical proficiency (bottom row). The vertical axes represent mean FA, and the horizontal axes represent subject age in years. The superior longitudinal fasciculus (SLF) and uncinate fasciculus (UF) tracts (top two rows) show a positive correlation between FA and age.

A simple linear regression of theFA values for bilateral SLF and UF in Table 1 yield the results shown in Figure 2. There is greater WM integrity in aging musicians in the bilateral SLF and bilateral UF.

In this experiment, the research hypothesis predicted positive slopes for four of the scatterplots (left and right SLF, left and right UF) and negative slopes for the left and right sagittal striata. Since the research hypothesis was directional, we did not conduct two-sided tests of whether the slopes were different from zero; instead, we predicted the signs of the slopes. Thus, we could halve the *p*-values from the two-sided tests. The *p*-values for each of the six regions of interest are as follows: LSLF, 0.3065; RSLF, 0.120; LUF, 0.1315; RUF, 0.197; LSS, 0.1435; RSS, 0.202. By applying the formula given above to all of the halved *p*-values of the regressions shown and then comparing the test statistic to a chi-squared distribution with 12 degrees of freedom (−2 Σ ^k^_i = 1_ ln(p_i_) = 20.994), the resulting pooled *p*-value is 0.050458, which is close to significance.

Based on previous research on cognitive brain reserve using DTI [33,34,35,36,37], we hypothesized that we would find increased WM integrity in FA values of two important tracts, SLF and UF, in lifelong musicians. We also hypothesized that the bilateral SS, a tract important for bilingualism, would not be impacted by musicianship and show loss as seen in healthy aging. Following our hypothesis, the superior longitudinal fasciculus (SLF) and uncinate fasciculus (UF) tracts show a positive correlation between FA and age in subjects with high musical proficiency, while FA decreases with age in the sagittal strata (SS) in the same subjects. Thus, the SS may be unrelated to musical proficiency.

The results are in keeping with our hypothesis. In future analysis, we will explore the WM integrity and FA values of bilateral SS and IFOF in bilinguals and multilinguals.

## 4. Discussion

The bilateral SLF and bilateral UF have been noted in the literature on cognitive reserve and bilingualism (Friederici, 2009; Luk et al., 2011; Madhavan et al., 2014; Pliatsikis et al., 2015) [10,11,44,45], while only the FA values of the bilateral SLF have been noted in the literature on cognitive reserve in musicianship, specifically with regard to relative and absolute pitch (Oechslin et al., 2010) [30]. Halwani et al. (2011) focused primarily on increased FA values in bilateral AF in musicians, including instrumentalists and singers [29]. In terms of the function of these white matter tracts in processing language, the dorsal–ventral pathway was often mentioned, but without further differentiation or explanation (Pliatsikas et al., 2015) [10].

In studies of cognitive reserve and healthy aging in musicians, there are no studies that focus specifically on the UF. The lesion–deficit DTI literature that included research involving musicians and music-based treatments identified the UF as important in both pitch perception in healthy subjects and music therapy [31,32]. There is a larger body of lesion–deficit studies that consider the bilateral UF and FA values, including visual memory delay (Riley et al., 2010; Diehl et al., 2008; McDonald et al., 2008) [46,47,48], naming impairment (Lu et al., 2002; Hamberher and Drake, 2006; Grabowski et al., 2001) and psychopathy (Craig et al., 2009) [49,50,51,52]. One possible point of interest emerges from the work of Duffau et al. (2009) on the possible role of the IFOF and UF in language processing with patients undergoing surgery (in the left anterior temporal lobe or orbitofrontal region) [53]. They were not able to confirm or reject the idea that the UF is essential in language processing.

While our scans did not show any systematic scanner-related errors, there is some research suggesting that all diffusion imaging may require additional correction (Krzyżak and Olejniczak, 2015; Borkowski and Krzyżak, 2018) [54,55]. Duke University BIAC is part of the Biomedical Informatics Research Network (BIRN) that examines potential system biases across vendors and has taken measures to address any concerns about systematic errors in DTI acquisition in Duke University Health System scanners (Helmer et al., 2016) [56].

The present study focuses on how musicianship may affect changes to WM integrity across the life cycle and change the trend of reduction in FA structures that are relevant in musicianship, specifically the SLF and UF. Previous research has shown that FA values in the SLF and UF show a significant decrease in FA values with age (Tang et al., 1997; Kochunov et al., 2007; Kochunov et al., 2011; Bennett et al., 2012; Giorgio et al., 2010; Westlye et al., 2010; Billiet et al., 2015; Rathee et al., 2016) [31,34,35,36,37,57,58,59].

Westlye et al. (2010) examined age changes in WM integrity in 430 healthy subjects between the ages of 8 and 85 years [35]. The SLF and UF were included bilaterally in their analysis of seven major WM tracts. Their findings showed a significant (*p* < 0.0001) decrease in FA values across whole-brain WM fiber tracts, and the maximum FA values were found at 29.1 years of age (2010: 2058–60) [35]. The age of maxima for SLF and UF were 28.8 and 28.6 years, respectively (2010: 2061). Rathee et al. (2016) also examined FA values for 177 healthy subjects and divided the subjects into three age groups: 20–40, 41–60 and 61–85 years old [37]. Whole-brain values showed a consistent decrease in FA values across each of the three age groups, and voxelwise FA values included a significant decrease in 22 regions (middle to oldest group), 26 regions (youngest to oldest group) and 4 regions (youngest to middle group). Furthermore, all FA values were lower in all regions between the oldest and youngest groups, and explicit reference to the SLF and SS showed significantly lower FA values in the oldest to youngest groups (2016: 14–15) [37].

As mentioned in the introductory section, the research focusing on cognitive reserve has shown that the process of WM integrity loss in normal aging may be slowed or changed by lifelong bilingualism and musicianship. Our study is unique in its requirement for active musicianship across the life cycle.

Our hypotheses looked specifically at the bilateral SLF and the bilateral UF. While previous studies on musicianship did not focus on the UF, given that it is one of the tracts with a later period of maturation and connects temporal and frontal lobe structures, it was included in our analysis. Our results support the research on cognitive reserve and show that the FA values for bilateral SLF and bilateral UF were greater in older musicians. These findings suggest that musicianship across the life cycle may change the expected decrease in FA values in these two subcortical tracts. A third tract, the bilateral SS, which includes the IFOF, a tract important in bi- and multilingualism, did not show the same effects.

### Limitations and Future Directions

The COVID-19 crisis resulted in access to Duke Hospital and the scanners being shut down, limiting the overall number of subjects and resulting in the lack of control subject data. Future directions include a replication of the results across a larger data set, nonmusician controls, investigation of the functional activations and connectivity through fMRI and other direct comparisons of musicians and bi- or multilinguals.

## 5. Conclusions

The present study has focused on lifelong professional musicians who play at least piano or violin. Our findings suggest that lifelong musicianship may contribute to WM integrity in the bilateral SLF and the bilateral UF in aging. While earlier research identified higher FA values in the right SLF (Li et al., 2014) [60], our findings show increases in bilateral SLF. The identification of higher FA values in the bilateral UF is also interesting and expands the examination of subcortical WM tracts that may be affected by lifelong musicianship. This study is a preliminary contribution to the growing body of research on behavioral cognitive reserve and suggests additional evidence that highly proficient musicianship may produce similar effects of higher FA values in certain WM tracts, while differing in others. Future studies that compare different types of musicianship (instrumentalists, vocalists, conductors) and bi- or multilinguals will provide new points of comparison between these groups and more specificity in defining the impact of cognitive reserve and changes to WM integrity in healthy aging.

## Figures and Tables

**Table 1:** Mean fractional anisotropy (FA) values with SD in parenthesis for each subject in specific ICBM-81 brain regions *.

Subject	LSLF	RSLF	LUF	RUF	LSS	RSS
1	0.471 (0.14)	0.471 (0.13)	0.472 (0.12)	0.472 (0.10)	0.531 (0.12)	0.528 (0.10)
2	0.512 (0.14)	0.503 (0.13)	0.505 (0.15)	0.530 (0.14)	0.560 (0.10)	0.580 (0.09)
3	0.472 (0.14)	0.488 (0.13)	0.456 (0.13)	0.493 (0.11)	0.540 (0.11)	0.544 (0.09)
4	0.462 (0.14)	0.457 (0.13)	0.426 (0.13)	0.470 (0.12)	0.580 (0.12)	0.562 (0.10)
5	0.465 (0.12)	0.482 (0.11)	0.411 (0.12)	0.465 (0.10)	0.571 (0.10)	0.581 (0.10)
6	0.481 (0.12)	0.490 (0.12)	0.441 (0.15)	0.480 (0.14)	0.593 (0.11)	0.615 (0.10)
7	0.475 (0.13)	0.488 (0.13)	0.467 (0.13)	0.468 (0.12)	0.569 (0.09)	0.586 (0.09)
8	0.483 (0.12)	0.475 (0.12)	0.436 (0.11)	0.476 (0.12)	0.563 (0.10)	0.599 (0.10)

* LSLF—left superior longitudinal fasciculus, RSLF—right superior longitudinal fasciculus, LUF—left uncinate fasciculus, RUF—right uncinate fasciculus, LSS—left sagittal striatum and RSS—right sagittal striatum. Regions were defined according to the ICBM-81 diffusion tensor imaging (DTI) atlas.
